# The role of the rapid molecular test (RMT) and the provision of a negative pressure mortuary room in reducing COVID-19 corpse handling protocol rejection: experiences with religious conservative groups

**DOI:** 10.1186/s41935-022-00267-5

**Published:** 2022-01-17

**Authors:** Arfi Syamsun, Hamsu Kadriyan, Ni Putu Sasmita Lestari, Ima Arum Lestarini, Arina Windri Rivarti, Agussalim Bukhari, Zikrul Haikal

**Affiliations:** 1 Faculty of Medicine, University of Mataram, Jalan Pendidikan 37, Mataram, 83125 Indonesia; 2 Mataram City General Hospital, Mataram, Indonesia; 3 Faculty of Medicine, Hasanuddin University, Makassar, Indonesia

**Keywords:** COVID-19, RMT, Negative pressure room, Corpse handling protocol, Religious conservative

## Abstract

**Background:**

This study explores the difference in COVID-19 corpse handling protocol rejection before and after the innovation in rapid molecular test (RMT) postmortem examination and providing negative pressure mortuary rooms. This study is a retrospective observational study. Each of the corpse’s immediate family was explained the procedure for handling the body based on the fatwa of the religious institution and the hospital’s standard operating system. The acceptance or rejection of the protocol, general characteristics of the corpse, and the reasons for refusal are documented.

**Results:**

From March to May 2020, there were 16 probable COVID-19 corpses and 3 confirmed COVID-19 corpses. Rejection of the COVID-19 corpse protocol occurred six times. The main reason for rejection is that the death might not necessarily be caused by COVID-19, the body’s handling in the hospital is not following religious law, and the negative stigma of COVID-19. From June to August 2020, there were 42 probable COVID-19 corpses and 49 confirmed COVID-19 corpses. Rejection of the COVID-19 corpse protocol occurred eight times. The most rejection reason is that the deceased families do not believe the deceased died because of COVID-19.

**Conclusions:**

The decline in the COVID-19 corpse protocol rejection has occurred after applying RMT and providing a negative pressure mortuary room. This decline proves that religious conservative groups can accept this innovation to reduce rejection on religious grounds.

## Background

Stigmatization and discrimination occurred almost all over the world at the beginning of the COVID-19 pandemic with various forms of stigma, including racial stigmatization, health workers and ambulance officers, groups belonging to certain religious sects, COVID-19 patients, and COVID-19 corpses (Villa et al. 2020; Bhanot et al. 2021). The stigma against the COVID-19 corpses and the stigma against the handling of corpses in hospitals need to be handled by forensicist (forensically trained pathologists) as a humanitarian duty to investigate sudden, unexplained, or unattended deaths and provide solutions to problems that occur in the process of corpse handling (Kementerian Kesehatan 2020; Mucheleng’anga and Himwaze 2020; Moretti et al. 2021). Appropriate stigma mitigation strategies in dealing with pandemics are critical to prevent the worsening of overall health risks and prevent social marginalization (Gronholm et al. 2021).

Stigma against COVID-19 corpses in Indonesia at the beginning of the pandemic consisted of three things: refusal to bury corpses in public cemeteries for fear of transmitting the virus, forced retrieval of corpses due to the corpse status was still suspected or probable and rejection of a COVID-19 diagnosis, the refusal of ritual burial of corpses in hospitals because the family of the corpse thinks the officers do not understand the procedures for religious funeral rituals (Sullivan 2020; Karmini and Milko 2020), and the lack of mortuary facilities (Windasari et al. 2020). The Indonesian government’s response is more focused on handling COVID-19 patients, but the government has less attention on handling COVID-19 corpses (Djalante et al. 2020). The response to handling corpses is not balanced with the fulfillment of adequate mortuary facilities, especially negative pressure mortuary room facilities, so that forensicist can perform infectious corpse handling, clinical autopsies, and embalming, especially in the Muslim and Hindu communities. Therefore, it is necessary to advocate for the government and hospital leaders.

The protocol of COVID-19 corpses will be more easily accepted if the status of the COVID-19 corpses is immediately confirmed and the common religious rituals for corpse handling can be accommodated (Baller et al. 2020; Kementerian Kesehatan 2020). RMT can be considered an alternative RT-PCR to confirm COVID-19 corpses because it can confirm COVID-19 more rapidly and has the same accuracy as RT-PCR in detecting viral nucleic acids. Furthermore, the negative pressure mortuary room aims to maintain the air pressure inside the room to be more negative than outside the room, lowers air escape to the surrounding area, and increases the air change rate. It will reduce the risk of viral transmission (Balocco and Lio 2011). In contrast to positive pressure rooms, which only prevent the transmission of viruses indoors, the air inside is always forced to flow out from the room to the surrounding environment by maintaining higher air pressure in the room. A negative pressure room system that maintains negative air pressure in the room provides protection from virus transmission to health workers, patients, and visitors in the surrounding environment. In addition, the air inside the room will also be taken out through a filtration system accompanied by ultraviolet radiation, which is able to sterilize and reduce microbes, including viruses. It helps protect health workers and their families when doing COVID-19 corpse handling (Al-Benna 2021). Thus, routine handling of the corpse such as bathing, wrapping in cloth, plastic, bags, coffins, and prayer rituals can be carried out properly (Baller et al. 2020). In the end, it can reduce the forced retrieval of COVID-19 bodies by the family (Anonim 2020; Wardhana 2020). This study aims to conduct a qualitative analysis of the effects of RMT postmortem and preparation of negative pressure rooms for COVID-19-positive corpses on the change of COVID-19 corpse handling protocol rejection.

## Methods

This research is a retrospective observational study carried out from 25 March 2020 to 31 August 2020 at the Mataram City Regional Hospital, six other private hospitals, and ten health centers in Mataram. The number of samples collected was 110 corpses in which the closest family to the corpse had given approval or rejection of the procedures for handling the COVID-19-positive corpses.

The period March 2020 to May 2020 is the first 3-month interval period, and the period June 2020 to August 2020 is the second 3-month interval period. Postmortem swab taking, RMT postmortem, and provision of a negative pressure mortuary room began in the second 3-month interval. Data on rejection and acceptance of COVID-19 corpse handling protocols before and after postmortem swab, postmortem RMT, and applying negative pressure mortuary room will be compared.

Each of the closest relatives of probable and confirmed COVID-19 corpses is given an explanation of the procedure for handling the corpse based on the fatwa of a religious institution and standard hospital operating procedures. Acceptance or rejection of the protocol is done in writing. The conservative religious status is assessed based on the reasons for rejection, both verbally and in writing, as outlined in the COVID-19 protocol rejection form. This research has been approved by the Faculty of Medicine research ethics committee, the University of Mataram, with approval letter No. 41/UN18.F7/ETIK/2021. Statistical analysis was performed using the chi-square test with a significance level of 0.05.

## Results

One hundred ten corpses were obtained, consisting of 52 (47.27%) confirmed COVID-19 corpses and 58 (52.72%) probable COVID-19 corpses. Postmortem swabs and RMT were undertaken on 33 (56.89%) probable COVID-19 corpses. The status of the COVID-19 corpses can be seen in Table 1.

**Table 1:** The status of the COVID-19 corpses

Corpses status	Period		Total
	**25 March 2020– 31 May 2020**	**1 June 2020–31 August 2020**	
Probable	16 (14.54%)	42 (38.18%)	58 (52.72%)
Confirmed	3 (2.72%)	49 (44.54%)	52 (47.27%)

Based on the status of rejection of the protocol for handling the COVID-19 corpse in the first 3-month interval, it shows that 6 (5.45%) families of the deceased still reject the protocol, 7 (6.36%) families accept the protocol, and 6 (5.45%) families accepted the protocol after negotiations with the hospital and security forces. Furthermore, in the second 3-month interval, 8 (7.27%) families continued to reject the protocol, 72 (65.45%) families accepted the protocol, and 11 (10%) families accepted after negotiations (Table 2).

**Table 2:** Family’s decision on COVID-19 corpse handling protocol

Family’s decision on COVID-19 corpse handling protocol	Period		Total	Significance
	**25 March 2020–31 May 2020**	**1 June 2020–31 August 2020**		
Reject	6 (5.45%)	8 (7.27%)	14	*P* = 0.015
Accept	13 (11.81%)	83 (75.45%)	96	

In the first 3-month interval, there were two rejections due to three reasons, including the family did not believe that COVID-19 caused the deceased death, the family considered the hospital morgue facilities to be inadequate for handling the corpse, and the family worried about the negative stigma of the corpse of COVID-19. In addition, there were also four rejections for two reasons: the family considered the hospital morgue facilities to be inadequate and worried about the negative stigma of COVID-19. Meanwhile, in the second 3-month interval, the family did not believe that the COVID-19 caused the deceased death is the rejection reason found in six cases.

## Discussion

The number of probable COVID-19 cases in the first 3-month interval was 16 (84.21%) from 19 corpses. This delay in diagnosis is because the hospital does not yet have a PCR or RMT device. It has to send nasopharyngeal or oropharyngeal swab samples to other cities such as Jakarta, where the RT-PCR results have only been out for about 7 days or more. Meanwhile, in the second 3-month interval, the number of probable COVID-19 cases who died was 42 (46.15%) of the 91 corpses. During this period, the hospital had PCR and RMT machines to obtain a diagnosis of COVID-19 immediately. The high number of probable cases is probably caused by the people who have brought the patient to the hospital if their condition is severe or critical. Hence, their status is still probable until the patient dies.

Examination of nasopharyngeal swab or oropharyngeal swab specimens using RT-PCR and RMT aims to improve the ability to diagnose COVID-19 (Afzal 2020; Anonim 2021). The RT-PCR test had limitations, including the fastest test results were known after 24 h, and running PCR was carried out after the minimum sample size had been met to save on the reagents use and other consumables. On the other hand, RMT has several advantages, namely (1) the same accuracy as RT-PCR in detecting viral nucleic acids, (2) test results can be obtained 1–2 h, and (3) this equipment is available in many districts/cities in Indonesia, which has been used in tuberculosis diagnostic examination programs so far (FDA 2020; GeneXpert 2021; Anonim 2021).

The COVID-19-positive corpses need to be carried out by postmortem swabs for social reasons, even though the postmortem swab is not required in the COVID-19 corpse handling protocol. The social reason is to make the status of the corpse clearer so that the family can accept the protocol for handling infectious bodies. Therefore, the RMT result can become a tool for negotiation when handling the COVID-19 corpse is needed. Then, the forcible removal of the COVID-19-positive corpse by the family can be avoided.

In this study, there were several reasons for refusal to handle the COVID-19 corpse in the first 3-month interval, namely the family did not believe that the deceased died because of COVID-19, the COVID-19 corpse handling protocol was not in accordance with religious law, and the negative stigma of the COVID-19-positive corpse was in the form of fear of being shunned by neighbors and family. The religious reasons are the basis for the rejection of the COVID-19 corpse handling protocol. In conservative religious communities, the new legal provisions related to handling infectious corpses are difficult to accept, causing social conflict between the community and the hospital. The conflict that cannot be handled will result in the forcible removal of the COVID-19-positive corpse.

The reason for refusing COVID-19 corpse handling in the second three-month interval is the family’s distrust of the diagnosis of COVID-19. This is most likely influenced by fake news or hoaxes spread around the COVID-19 outbreak, such as the COVID-19 conspiracy theory and the allegation that the COVID-19 examination and corpse handling is only for the benefit of the hospital business. This negative prejudice has caused distrust in the diagnosis of COVID-19.

According to Hinduism, death is a series of transition processes to another life (reincarnation). A ritual of respect is needed so that the soul (atman) achieves a new, better life. There are differences in Hindu religious rituals in India, which are divided into 15 regions. Therefore, it is crucial to assess variations in practices and death beliefs among different groups (Gupta 2011). The majority of Hindu sects in Indonesia are Balinese Hindus. However, the religious philosophy is the same as in India, such as reincarnation, karma, dharma, ahimsa, atman, and moksha (McDaniel 2010; Yoga Segara 2018). However, in the practice of religious rituals, there are some differences. One of the differences in the funeral rituals for the corpses of Hindus in Indonesia is that the cremation process/burial must wait for a good day according to the instructions of Hindu religious leaders (pedanda). Sometimes, the process of waiting for the auspicious day takes up to a month or even more, especially since the corpse is a religious figure or community figure. Thus, embalming is a must so that the body remains intact until the day of the funeral.

The embalming process is not recommended according to the WHO protocol, but if it has to be done in forensic cases, it must be done in a negative pressure room (Kementerian Kesehatan 2020; WHO 2020). Unfortunately, most morgues in Indonesia do not own this facility, including hospitals in Lombok, Indonesia. This facility is only available in the isolation treatment room for COVID-19 patients.

Funeral rituals for adherents of Islam are bathing, wearing the shroud, praying the body, and delivering the funeral as soon as possible after death (Sarhill et al. 2001; Gatrad and Sheikh 2002). Local elements included in the ritual are bathing the corpse carried out by the closest family accompanied by religious leaders who are usually old to lead prayers, flowing clean water to clean all dirt, both on the surface of the body and dirt in the body such as feces, urine, and phlegm by pressing stomach and chest, and the use of a white shroud without plastic; the funeral does not use a coffin, and the cheeks of the corpse rest on the ground.

The ritual is prohibited in the COVID-19 protocol because it can cause virus transmission to officers and families who carry out the ritual (Kementerian Kesehatan 2020; WHO 2020). However, if it is carried out in a negative pressure room and complete PPE, the entire series of bathing and the use of the shroud can be carried out. This innovation is an acceptable alternative for most religious conservative groups.

## Conclusions

Taking postmortem swabs, examining RMT, providing negative pressure mortuary rooms in hospitals, and involving families in corpse handling are alternatives that most conservative religious groups can accept, thereby reducing the number of cases of the forcible removal of COVID-19-positive corpses.

## Data Availability

The datasets used and/or analyzed during the current study are available from the corresponding author on reasonable request.

## Abbreviations

RMT: Rapid molecular test
ARI: Acute respiratory infection
WHO: World Health Organization
PCR: Polymerase chain reaction
RT-PCR: Real-time polymerase chain reaction
